# Adolescents and Young Adults with Type 1 Diabetes Present Changes in Arterial Compliance and Resistance and Increased Arterial Stiffness

**DOI:** 10.1155/2024/9919121

**Published:** 2024-04-17

**Authors:** Igor A. Carvalho-Ribeiro, Letícia C. F. Cunha, Lorena R. Ribeiro, Matheus N. Lima, Breno A. Ferreira-Silva, Juliana S. Rajão, Juliana C. Bittencourt, Juliana F. A. Pinheiro, Maria da Glória Rodrigues-Machado

**Affiliations:** ^1^Faculty of Medical Sciences of Minas Gerais, Belo Horizonte, Minas Gerais, Brazil; ^2^Endocrinology Service, Hospital das Clínicas, Federal University of Minas Gerais, Belo Horizonte, Minas Gerais, Brazil; ^3^Iria Specialized Consultation Center, Contagem, Minas Gerais, Brazil

## Abstract

**Introduction:**

Cardiovascular disease (CVD) is highly prevalent in patients with type 1 diabetes (T1DM) and is responsible for a significant reduction in life expectancy.

**Objective:**

To compare the arterial stiffness indices, arterial compliance and vascular resistance assessed centrally and peripherally between healthy adolescents and young adults (CTRL group) and those with T1DM.

**Methods:**

This is an observational cross-sectional study involving 90 adolescents and young adults, with half of them being considered healthy (*n* = 45) and the other half with T1DM (*n* = 45), matched by age and sex. Cardiovascular parameters were evaluated using the oscillometric method of brachial artery pressure assessment for a noninvasive estimation of central arterial pressures.

**Results:**

Weight and body mass index were significantly higher in the T1DM group. AIx@75 was significantly higher in the T1DM group (24.96% ± 8.88%) compared to the CTRL (20.16% ± 9.95%). Peripheral and central arterial compliance were significantly lower in the T1DM group (0.79 ± 0.21; 1.16 ± 0.27 ml/m^2^/mmHg) compared to the CTRL (0.98 ± 0.32; 1.47 ± 0.61 ml/m^2^/mmHg). Peripheral and central vascular resistance were significantly higher in the T1DM group (1.32 ± 0.32; 0.91 ± 0.21 mmHg/ml/m^2^) compared to the CTRL (1.11 ± 0.30; 0.75 ± 0.22 mmHg/ml/m^2^).

**Conclusion:**

Our data confirm premature aging of the vascular system in adolescents and young adults with T1DM and extend our knowledge by showing important changes in central and peripheral hemodynamics indices.

## 1. Introduction

Diabetes mellitus is a serious chronic disease characterized by elevated blood glucose concentrations related to the effects of abnormal *β*-cell biology on insulin action [1]. The most common types are type 1 diabetes mellitus (T1DM) and type 2 (T2DM). Symptoms of T1DM generally appear in childhood and adolescence, in which, it is necessary to begin glycemic control due to the lack of a cure so far [2]. The International Diabetes Federation (IDF) estimated that 451 million adults were living with diabetes worldwide in 2017, with a projected increase to 693 million by 2,045 if no effective prevention methods are adopted [3]. The prevalence of T1DM and T2DM among children and adolescents has also increased and estimates of children and adolescents under 20 with T1DM now exceed one million [1]. The glycemic control is effective in preventing macrovascular diseases. Nonetheless, chronicity, hyperglycemic events, hypercholesterolemia, and inflammation are factors which may contribute to the risk of atherosclerosis in this population [4]. These changes compromise important organs and trigger events such as cardiac dysfunction, myocardial ischemia, and peripheral vascular disease that can lead to morbidity and mortality in this population [2].

Arterial stiffness can be assessed using different methods and different indices; however, three arterial stiffness indices can be identified by evaluating the aortic pulse wave: the augmentation index corrected for the heart rate of 75 bpm (AIx@75), the central aortic pulse pressure (PPc), and the central systolic blood pressure (SBPc). The aortic pulse wave can be generated easily, accurately, and noninvasively using a validated transfer function. The aortic pulse wave is composed of the ejection wave and reflection wave. The ejection wave is generated with each heartbeat. It travels through the arterial bed until it encounters peripheral resistance at bifurcation points or in any segment where there is flow discontinuity. From then on, the reflected wave is transmitted backward [5]. In addition to the conduction function of the large arteries, they are also responsible for instantly accommodating the blood volume ejected from the left ventricle. The elastic recoil of the arterial wall that occurs during diastole is responsible for maintaining diastolic flow in various tissues, and this process is fundamental for maintaining myocardial and cerebral perfusion throughout the cardiac cycle [6].

Our group recently demonstrated that the AIx@75, the augmentation pressure, and the reflection coefficient, as well as the PPc are significantly higher in a group of patients with T1DM compared to healthy controls, demonstrating accelerated vascular aging in this population [7]. Telezamaz et al. [8] showed that arterial stiffness indices correlate with glycated hemoglobin (HbA1c) in children and adolescents with T1DM. However, arterial compliance (systolic volume index/pulse pressure), vascular resistance (pulse pressure/systolic volume index) were not evaluated in adolescents and young adults with T1DM. Peripheral PP is an independent predictor of future left ventricular mass, vascular hypertrophy, and cardiovascular morbidity and mortality. A high PP results, at least partly, from stiffening of the arterial tree. Although PP alone is used as a crude index of arterial stiffness or, conversely, arterial compliance, arterial stiffness is defined as the relationship between changes in pressure and volume, and vice versa for arterial compliance. Therefore, considering volume variation may improve the estimation of vascular wall properties [9].

The objective of the present study was to compare the arterial stiffness indices, peripheral and central arterial pressures, arterial compliance, and vascular resistance between healthy adolescents and young adults and those with T1DM. Considering that patients with T1DM present premature vascular aging, our hypothesis is that vascular resistance will be increased and arterial compliance will be decreased in T1DM patients. Greater knowledge of the effects of T1DM in this young population can contribute to formulating important public health policies.

## 2. Materials and Methods

This is an observational cross-sectional study with the participation of 90 adolescents and young adults between 13 and 22 years old of both sexes. The T1DM group consisted of 45 adolescents and young adults diagnosed with T1DM. The control group (*n* = 45) was matched for age and sex with the T1DM group, using a previous database of adolescents and young adults.

The variables investigated in the control and T1DM groups were anthropometric data, vascular and hemodynamic parameters, and arterial stiffness indices. Data on comorbidities and illness duration were collected from the medical records of patients with T1DM.

### 2.1. Anthropometric Assessment

Anthropometry was collected as recommended by the World Health Organization [10]. Weight and height were measured, and body mass index (BMI) was calculated as the ratio between weight (kg) and height squared (m^2^). The participants were classified as eutrophic, overweight, and obese. BMI curves by sex and age were used to diagnose obesity in adolescent participants and evaluated according to the *Z* score or percentile (*P*). Percentiles greater than or equal to 97 or greater than or equal to *Z* + 2 were considered obese [11]. Patients over 18 years of age with a BMI greater than 30 kg/m^2^ were considered obese.

### 2.2. Assessment of Cardiovascular Parameters

Cardiovascular parameters were evaluated using the Mobil-O-Graph® (IEM, Stolberg, Germany), validated in comparative studies with invasive [12], and noninvasive methods [13] for measuring pulse wave velocity (PWV). This device uses an oscillometric method of evaluating brachial artery pressure to noninvasively estimate central arterial pressures (systolic blood pressure—SBPc, diastolic—DBPc, and pulse pressure—PPc). To do so, an ARCSolver algorithm (Austrian Institute of Technology, Vienna) [14] and a high-fidelity sensor (MPX5050; Freescale, Tempe, AZ, USA) are incorporated into the common cuff. The ARCSolver software program includes an algorithm to check signal quality on a scale of 1–4. Only excellent or good quality results which are labeled in grades 1 and 2 were considered in this study, which, respectively, includes more than 80% or more than 50% of the cardiac cycles recorded during signal acquisition [15].

Upper arm circumference was measured at the midpoint between the acromion and olecranon to determine the appropriate cuff size. The participants remained seated with their feet flat on the floor during the cardiovascular parameter assessment, in a calm and peaceful place after a 20-min rest. The left upper limb remained supported and relaxed at heart level during the measurement.

Arterial stiffness indices, PWV and AIx@75, were evaluated in accordance with previous studies by our group [7, 16–18]. PWV was determined using a mathematical model considering several parameters in the pulse wave and wave separation analysis. AIx@75 was calculated through the pressure difference between the peak of the reflection wave (P2) and the peak of the incident wave (P1), expressed as the percentage of PPc (AIx@75 = (P2–P1)/PPc × 100). The hemodynamic parameters evaluated were stroke volume (SV), cardiac output (CO), cardiac index (CI), and heart rate (HR). Arterial compliance (systolic volume index/pulse pressure) [19] vascular resistance (pulse pressure/systolic volume index) [20] were analyzed from the hemodynamic parameters. The stroke volume index was calculated by the stroke volume to body surface ratio [21].

Three measurements were taken, with the average between them being considered for analysis. Patients were instructed to abstain from physical activity and consumption of caffeine and chocolate, alcohol, and nicotine on the day of the research [22].

### 2.3. Sample Calculation

The sample size was calculated considering the comparison of two independent groups in the *G* Power 3.1.9.6 program setting a medium effect size, *d* = 0.60 (based on the article by Duarte et al. [7], considering the difference between the hemodynamic parameters of the group of diabetic patients and controls), significance level (alpha) at 0.05 and power (beta) at 80%. The sample size calculation determined that each group should consist of 45 patients resulting in a total sample of 90 patients.

### 2.4. Statistical Analysis

Continuous variables were expressed as mean ± SD. Data normality was assessed using the Shapiro–Wilk test. Continuous variables were compared using the Student's *t*-test (parametric variables) and the Mann–Whitney test (nonparametric variables). Categorical variables were described as percentages. Correlations between arterial stiffness indices and the different variables studied were performed using the Pearson's or Spearman's correlation coefficient, when indicated. To evaluate the controlled effect of T1DM on PWV, a linear regression model was built with groups, BMI and HR as covariates. A similar approach was done for AIx@75, considering as covariates the groups, mean arterial pressure, and height. The analysis was developed using the GraphPad Prism program (version 5.0; GraphPad Software, Inc., La Jolla, California). The significance level adopted was 5%.

## 3. Results

The disease duration was 8.96 ± 3.13 years and the average HbAIc was 9.82% ± 2.19%, demonstrating inadequate glycemic control. HbAIc was assessed within a maximum interval of 30 days from the collection of cardiovascular parameters.

Table 1 presents the anthropometric data of the participants. The T1DM group had significantly higher weight and BMI than the control group.

Cardiovascular parameters are presented in Table 2. Central and peripheral vascular pressures did not differ between groups. It was observed that heart rate was significantly higher in the T1DM group. Arterial stiffness assessed by AIx@75 and reflection coefficient were significantly higher in the T1DM group. In the linear regression model for AIx@75 considering the groups, height, and MAP as covariates, we observed that T1DM patients had an increase of 4.05 (0.06–8.04) compared to controls (*P*=0.047). AIx@75 correlated with HbAIc in the T1DM group. The PWV did not differ between the groups.

**Figure 1:**
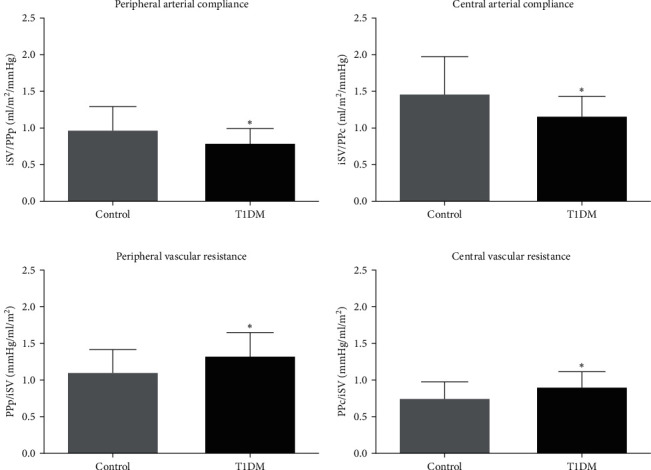
Peripheral and central arterial compliance (iSV/PPp and iSV/PPc) and vascular resistance (PPp/iSV and PPc/iSV). PPc, central pulse pressure; PPp, peripheral pulse pressure; iSV, stroke volume index.  ^*∗*^*P* < 0.05. (a) Peripheral arterial compliance, (b) central arterial compliance, (c) peripheral vascular resistance, and (d) central vascular resistance.

Peripheral and central arterial compliance, respectively, assessed by the iSV/PPp and iSV/PPc ratios, were significantly lower in the T1DM group when compared to the control group. Peripheral and central vascular resistance, respectively, assessed by the PPp/iSVand PPc/iSV ratios, were significantly higher in the T1DM group (Figure 1). Vascular resistance was significantly higher (*P* < 0.0001) in the periphery compared to the central region in the control and T1DM groups. On the other hand, arterial compliance was significantly higher (*P* < 0.0001) in the central region in both groups.

Celiac disease, Hashimoto's thyroiditis, primary amenorrhea, and dyslipidemia were the most prevalent comorbidities.

## 4. Discussion

This study compared the cardiovascular parameters and anthropometric data of healthy adolescents and young adults to those with T1DM and showed for the first time a reduction in arterial compliance and an increase in vascular resistance assessed in the central and peripheral vasculature in the group with DM1 compared to control group. The study of arterial stiffness in young people with T1DM enables better understanding of the initial stages of vascular disease using noninvasive techniques.

Arterial blood pressure is dependent on interactions between the heart and arteries. Resistive and pulsatile components of arterial load can be assessed by systemic vascular resistance (microvascular property) and the ratio of stroke volume to pulse pressure (a surrogate of total arterial compliance), respectively [23]. In the present study, we evaluated the hemodynamic indices of arterial compliance and vascular resistance in the central and peripheral regions for the first time. Hemodynamic assessment of different regions is important due to increased central stiffness has been associated with left ventricular hypertrophy [24], coronary disease, stroke, and mortality [25], and peripheral stiffness presents a greater risk for peripheral vascular disease [26]. Arterial compliance was calculated by the relationship between the stroke volume index and central (central arterial compliance) or peripheral (peripheral arterial compliance) pulse pressure. Our results showed that arterial compliance was significantly higher in the central region than peripheral region in both groups and were, respectively, 19.39% and 21.09% lower T1DM in relation to the control group. Lilly et al. [23] demonstrated a significant relationship between arterial compliance and subsequent cardiovascular events in an adults healthy population (45–84 years) without known cardiovascular disease.

Reduced arterial compliance assessed by Doppler echocardiography in adults individuals with T2DM was associated with overall mortality independent of blood pressure level. This result demonstrated that arterial compliance is more sensitive to the loss of vascular integrity observed in diabetic patients than simple blood pressure measurements [27]. It is important to highlight that pulse pressure, measured centrally or peripherally, was similar in both groups and that the use of the relationship between stroke volume index and pulse pressure can detect vascular dysfunction early.

Similar to arterial compliance, vascular resistance was calculated by the relationship between central or peripheral pulse pressure and the stroke volume index (central vascular resistance) or peripheral (peripheral vascular resistance). Vascular resistance levels were significantly higher in the periphery compared to the central region in both groups and showed an increase of 18.92% and 21.33% in T1DM, respectively, in relation to the control group. These results suggest overall impairment of vascular health in this population.

Greater understanding of the anatomical distribution of arterial stiffness will enable identifying patients at higher cardiovascular risk and the adaptation of therapy to prevent vascular damage in target organs. Fagard et al. [9] assessed retrospectively the prognostic power of the vascular resistance for cardiovascular events and mortality in patients with uncomplicated hypertension. The authors observed that for each 0.75 mmHg/ml/m^2^ increase in the PP-to-iSV ratio was independently associated with a 79% increase in the risk of a cardiovascular event and a 2.05-fold greater risk of all-cause mortality.

Similar to our results, studies show that the AIx@75 [7, 28] and the reflection coefficient [7] are significantly higher in the T1DM group compared to the control group. AIx@75 represents the combination of reflected waves with the wave propagated directly into the aorta [29]. The magnitude of the reflection wave [30] is measured by the reflection coefficient, meaning the ratio between the amplitude of the reflection and the ejection waves, considered as one of the main determinants of AIx@75.

In the present study, we used AIx corrected for a heart rate of 75 bpm (AIx@75) due to the significant, inverse, and linear relationship between AIx and heart rate. The increase in heart rate decreases the absolute duration of systole, shifting the reflected wave into diastole, reducing the AIx. Wilkinson et al. [31] demonstrated that for every increase of 10 bpm there is a 4% drop in AIx. In the present study, heart rate and AIx@75 were, respectively, 11.1% and 23.81% higher in the T1DM group compared to the control group.

In this study, we observed that AIx@75 correlated with HbAIc in the T1DM group. The disease duration was 8.96 ± 3.13 years and the average HbAIc was 9.82% ± 2.20%, demonstrating inadequate glycemic control. Similar results were found by Urbina et al. [32] in a sample of 1,819 adolescents and young people (17.6 ± 4.5 years) and with a similar illness duration to our study (7.8 ± 1.9 years). Urbina et al. [32] showed that arterial parameters were higher in participants with HbAIc ≥ 9% and PWV was higher with lower insulin sensitivity or longer duration of diabetes mellitus. In the present study, HbAIc was not associated with a longer duration of the disease. We performed a new analysis categorizing the sample with patients HbAIc ≥ 9% (*n* = 23) and HbAIc < 9% (*n* = 22) and evaluated the association of patients with HbAIc ≥ 9% with central and peripheral vascular pressures and duration of T1DM. We did not find any association between these variables. We also analyzed the association of PWV with the duration of T1DM and observed no association between these variables. As the population involved and the duration of T1DM are similar, a possible explanation for this difference in results between studies could be the sample size.

Despite the higher AIx@75 values in the T1DM group, PWV was similar between the groups in the present study. Studies show that these measurements of arterial stiffness are not interchangeable. AIx@75 depends on the propagation speed of the wave, the amplitude of the reflected wave, the reflection points, and the ventricular ejection duration and pattern, especially regarding to changes in heart rate and ventricular contractility [33]. In contrast, PWV evaluates the wave propagation speed and represents intrinsic arterial stiffness. The results of studies that evaluate PWV are controversial.

The SEARCH study longitudinally evaluated 298 young T1DM in 2013 and showed that this population presented an increase in PWV. This increase was progressive over time and associated with other risk factors, such as central adiposity or dyslipidemia, as well as worse glycemic control [34]. Putarek et al. [35] compared PWV in 68 obese adolescents, 42 with T1DM, and 38 controls. The authors observed that the T1DM group had the highest arterial stiffness, demonstrating that T1DM affects the vasculature in adolescence more than obesity. Moreover, disease duration in adolescents with T1DM was the greatest determinant of arterial stiffness, followed by body mass index, systolic and diastolic blood pressures, and HbA1c.

The prevalence of obesity has increased rapidly in the general population and among patients with diabetes [36]. A cohort of children with T1DM showed a marked increase in both overweight patients, from 28.6% to 47%, and obese patients, from 3.4% to 22.7%, over an 18-year period [37]. BMI was significantly higher in the T1DM group than the control group in the present study and was associated with vascular dysfunction. BMI negatively correlated with central arterial compliance and positively with central and peripheral vascular resistance.

## 5. Conclusions

This study showed for the first time reductions in arterial compliance and increased vascular resistance assessed centrally and peripherally in the TIDM group compared to control group. In addition, we observed AIx@75 was significantly higher in the T1DM group compared to the CTRL. The results of this study reinforce the concept of arterial aging accelerated by T1DM and extend our knowledge by showing important changes in central and peripheral hemodynamics.

## Figures and Tables

**Table 1:** Sample characterization.

Variables	Control, *N* = 45	T1DM, *N* = 45	*P*-value
Age (years)	16.60 ± 2.65	16.62 ± 2.56	0.9513^w^
Sex (M/F)	(19/26)	(19/26)	—
Weight (kg)	54.15 ± 13.12	59.98 ± 13.34	0.0394^t^
Height (m)	1.75 ± 0.93	1.61 ± 0.09	0.3657^w^
Body surface (cm^2^)	1.54 ± 0.29	1.62 ± 0.21	0.1764^w^
Body mass index (kg/m^2^)	20.36 ± 2.58	22.95 ± 4.25	0.0021^w^
HbA1c (%)	—	9.82 ± 2.19	—
Disease duration (years)	—	8.96 ± 3.13	—
Classification of associated diseases (%)			
	—	Celiac disease (15.55) Hashimoto's thyroiditis (15.55) Dyslipidemia (15.55) Primary amenorrhea (15.55) Autism (4.44) Epilepsy (2.22) ADD (2.22) Falsiform anemia (2.22) Cardiac arrhythmia (2.22)	—
Weight classification (%)			
	Eutrophic (91)	Eutrophic (71)	—
	Overweight (9)	Overweight (20)	—
	—	Obesity (9)	—

Data are represented as mean ± SD. *P*-values refer to the following: *w* = Mann–Whitney test and *t* = Student's *t*-test for independent samples; ADD, attention deficit disorder.

**Table 2:** Peripheral and central blood pressure, hemodynamic parameters, and arterial stiffness indexes between the control and diabetes groups.

Variables	Control, *N =* 45	T1DM, *N =* 45	*P*-value
Peripheral blood pressure			
SBPp (mmHg)	113.7 ± 9.79	118.20 ± 15.26	0.3710^w^
DBPp (mmHg)	70.55 ± 8.58	74.25 ± 12.10	0.3247^w^
MAPp (mmHg)	90.21 ± 8.26	94.04 ± 12.11	0.2037^w^
PPp (mmHg)	43.10 ± 7.36	43.46 ± 12.82	0.8940^w^
Central blood pressure (aorta)			
SBPc (mmHg)	101.2 ± 9.10	105.9 ± 14.74	0.1532^w^
DBPc (mmHg)	72.12 ± 8.51	76.34 ± 12.66	0.2585^w^
MAPp (mmHg)	81.82 ± 8.22	86.20 ± 12.67	0.1676^w^
PPc (mmHg)	29.09 ± 6.12	30.53 ± 8.19	0.5914^w^
Hemodynamic parameters			
Systolic volume (ml)	61.50 ± 15.30	55.60 ± 14.14	0.0538^w^
Cardiac output (l/min)	4.69 ± 0.59	4.68 ± 0.64	0.6805^w^
Cardiac index (l/min/m^2^)	3.07 ± 0.45	2.95 ± 0.50	0.1163^w^
Heart rate (bpm)	**78.11 ± 13.75**	**87.10 ± 17.70**	**0.0086** ^**t**^
Arterial stiffness			
Alx@75 (%)	**20.16 ± 9.95**	**24.96 ± 8.88**	**0.0177** ^**t**^
Reflection coefficient (%)	**55.82 ± 7.09**	**58.63 ± 9.23**	**0.0373** ^**w**^
Augmentation pressure (mmHg)	5.64 ± 2.46	7.34 ± 4.67	0.1199^w^
Pulse wave velocity (m/s)	4.64 ± 0.33	4.77 ± 0.54	0.4382^w^

Data are represented as mean ± SD. SBPp, peripheral systolic blood pressure; DBPp, peripheral diastolic blood pressure; MAP, mean arterial pressure; PPp, peripheral pulse pressure; SBPc, central systolic blood pressure; DBPc, central diastolic blood pressure; PPc, central pulse pressure; SV, Stroke volume; AIx@75, augmentation index corrected for heart rate of 75 bpm. *w* = Mann–Whitney test and *t* = Student's *t*-test for independent samples. Bold values indicate the variables are significant.

## Data Availability

All data generated or analyzed during this study are included in this published article.
